# Balancing the good and the bad: controlling immune-related adverse events versus anti-tumor responses in cancer patients treated with immune checkpoint inhibitors

**DOI:** 10.1093/immadv/ltac008

**Published:** 2022-04-08

**Authors:** Guilherme Ferreira de Britto Evangelista, Amanda Braga Figueiredo, Milton José de Barros e Silva, Kenneth J Gollob

**Affiliations:** Translational Immuno-oncology Laboratory, Hospital Israelita Albert Einstein, São Paulo, SP, Brazil; Center for Research in Immuno-oncology (CRIO), Hospital Israelita Albert Einstein, São Paulo, SP, Brazil; Translational Immuno-oncology Group, International Center for Research, A.C.Camargo Cancer Center, São Paulo, SP, Brazil; Translational Immuno-oncology Laboratory, Hospital Israelita Albert Einstein, São Paulo, SP, Brazil; Center for Research in Immuno-oncology (CRIO), Hospital Israelita Albert Einstein, São Paulo, SP, Brazil; Clinical Oncology Department, A.C.Camargo Cancer Center, São Paulo, SP, Brazil; Translational Immuno-oncology Laboratory, Hospital Israelita Albert Einstein, São Paulo, SP, Brazil; Center for Research in Immuno-oncology (CRIO), Hospital Israelita Albert Einstein, São Paulo, SP, Brazil

**Keywords:** cancer immunotherapy, toxicity, T-cells, immune mechanisms, autoimmunity, adverse events, checkpoint inhibitors

## Abstract

Immune checkpoint inhibitors (ICI) have provided new hope for cancer patients, and in particular for patients with tumors that are immunologically active and classified as hot tumors. These tumors express antigenic and tumor microenvironment (TME) characteristics that make them potential candidates for therapy with checkpoint inhibitors that aim to reactivate the immune response such as anti-PD-1 and anti-CTLA-4. Examples of potentially responsive cancers are, melanoma, non-small cell lung cancer and several other metastatic or unresectable tumors with genetic instability: DNA mismatch repair deficiency (dMMR), microsatellite instability-high (MSI-H), or with a high tumor mutational burden (TMB). Immunotherapy using checkpoint inhibitors is typically associated with adverse events (AEs) that are milder than those with chemotherapy. However, a significant percentage of patients develop short-term immune-related AEs (irAEs) which range from mild (~70%) to severe cases (~13%) that can lead to modifications of the checkpoint inhibitor therapy and in some cases, death. While some studies have investigated immune mechanisms behind the development of irAEs, much more research is needed to understand the mechanisms and to develop interventions that could attenuate severe irAEs, while maintaining the anti-tumor response intact. Moreover, studies to identify biomarkers that can predict the likelihood of a patient developing severe irAEs would be of great clinical importance. Here we discuss some of the clinical ramifications of irAEs, potential immune mechanisms behind their development and studies that have investigated potentially useful biomarkers of irAEs development.

## Introduction

Immune checkpoint inhibitors (ICI) have ushered in a new era of therapeutic options for cancer patients, offering new hope to many patients. Immunotherapy by reactivation of suppressed anti-tumor responses has proven to be highly effective in many tumors. ICIs have revolutionized the treatment of several tumor types, some of them reaching never before seen survival rates, in particular those that have a permissive tumor microenvironment (TME), characterized by genetic instability (DNA mismatch repair deficiency (dMMR) or microsatellite instability-high (MSI-H)), or with a high tumor mutational burden (TMB) [1–3]. However, one of the major challenges in clinical practice dealing with ICIs is the adequate management of immune-related side effects, ranging from simple observation with no need for treatment interruption, to situations of imminent risk of death, demanding severe immunosuppression [4].

While adverse events (AEs) are generally milder in patients treated with ICI therapies than those treated with chemotherapy, recent studies have shown that up to 69% of patients display short term or acute side effects and of these, 13% were severe or deadly. Moreover, up to 43% of patients presented long term or chronic side effects lasting 3 months or more after cessation of ICI therapy [5]. The balance between the beneficial effects of ICI therapy to induce an effective anti-tumor response and the undesired effects of inducing auto-aggressive immune responses is key for determining if a given patient will experience an overall benefit from the therapy or not.

**Figure 1:**
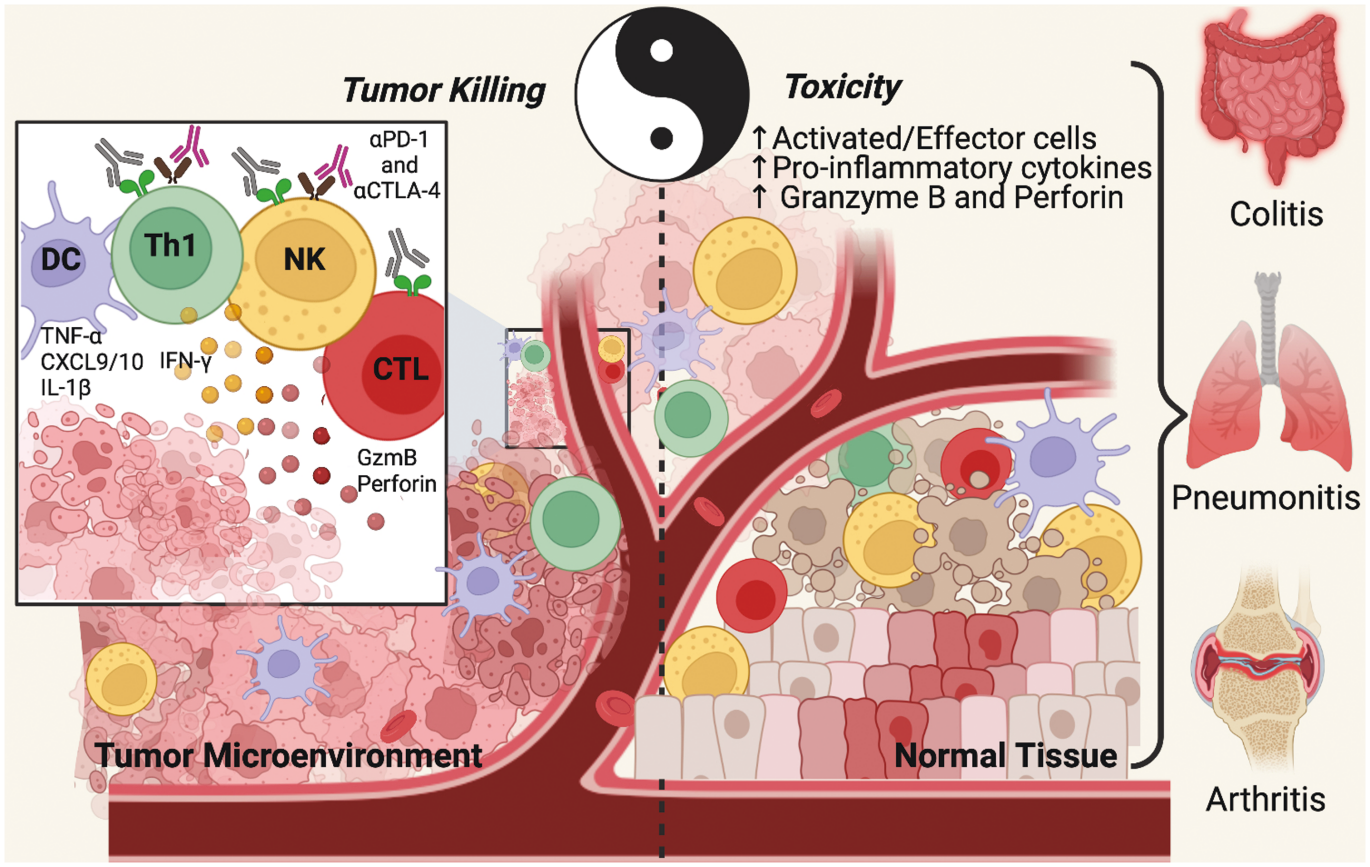
The Ying and Yang of cancer immune checkpoint inhibitory therapy. Once the immune checkpoints are inhibited by the monoclonal antibodies (αPD-1 and αCTLA-4) the anti-tumor response is released and tumor killing is expected to increase (left side). As a basic representation, dendritic cells (DC) in the tumor microenvironment present antigens to T cells (T helper *or* cytotoxic cells) activating them and also producing many pro-inflammatory cytokines (TNF-α, CXCL9/10, IL-1β, IL-12, and others) that reinforces the effector activity of immune cells in general. Focusing on tumor killing, Th1 cells produce IFN-γ that acts as a key factor contributing to activation and activity of NK and cytotoxic cells (CTL) that directly kill the tumor through granzyme B and perforin. On the other hand (right side), even though the checkpoint inhibitors released the anti-tumor response, from a systemic point of view, the increase of pro-inflammatory mechanisms at the tumor site and systemically can impact other immune responses. Thus, the increase of activated and effector cells as well as the increase in pro-inflammatory cytokines and perforin/granzyme B production can lead to normal tissue inflammation and consequently damage in some cases. This outcome is expressed as toxicity of immune checkpoints inhibitors, also known as immune-related adverse events (irAEs), and can be directed to many organs causing colitis, pneumonitis, arthritis, and other inflammatory conditions. Figure designed using Biorender.com.

One of the most urgent issues is understanding the immune mechanisms behind development of the anti-tumor response versus those responsible for induction of irAEs to see if specific mechanisms can be identified and used as therapeutic targets for reducing the severity or frequency of irAEs, while maintaining the anti-tumor response intact (Fig. 1). The two are clearly related given that patients treated with ICIs subsequently develop irAEs. However, it is possible that distinct characteristics behind the generation of the beneficial anti-tumor response versus the detrimental irAEs can be identified either at the level of immune cell activation states, functional potential, or exhaustion potential, as well as at the level of antigen specificity and dominant clonal T and/or B-cell responses. Any of these differential mechanisms could open the way for development of novel adjuvant therapies or identification of clinically useful biomarkers that would allow the clinician to predict which patients will most benefit from ICI therapies.

## Clinical implications of AEs in ICI therapy of cancer

The ImmunoCancer International Registry, which is a data-sharing multidisciplinary network from 18 countries focused on immune-related adverse events (irAE), has shown an accumulated annual number of nearly 13 000 irAE cases reported in 2018 and it has been increasing exponentially due to new indications of ICI alone or in combination with other agents, as well as increasing use of these therapies globally [6]. Therefore, attention to the impact of irAEs on patient safety and success of the treatment is essential.

An elegant systematic review and network meta-analysis of 36 phase II/III trials providing a complete toxicity profile of the main ICI drugs (nivolumab, pembrolizumab, ipilimumab, tremelimumab, and atezolizumab) has shown a pooled incidence for all AEs ranging between 54% and 76% [7]. Analyzing irAE incidence data from the pivotal ICI studies in oncology, rates of grade 3 or 4 irAE (severe or life-threatening), are approximately 10–16% for anti-PD-1/L1, 20–31% anti-CTLA-4, and 40–55% for the combination of anti-PD-1 and anti-CTLA-4 [8–11]. The global mortality rate associated with ICI is estimated to be up to 1.23% for combination but it can reach extremely high numbers depending on the type of side effect, as seen by the 48% of mortality in patients who develop myocarditis [12, 13].

Overall, any organ or system can be affected by these autoimmune side effects. However, the most frequently reported systems are dermatologic, gastrointestinal, endocrine, hepatic, pulmonary, and renal [14]. The median onset of symptoms depends on the organ or system affected, in general within 2–16 weeks from the beginning of treatment, the majority of them (85%) occurring during the first 16 weeks [15]. However, irAEs can occur after this period, and cases of symptoms emerging more than one year after completion of therapy have been described [16].Occasionally, irAEs may lead to irreversible organ damage, such as fibrotic pulmonary damage, non-resolved xerostomia, vitiligo, alopecia, colon perforation and, up to 20% of cases may lead to some endocrine insufficiency requiring lifelong hormone replacement [17–20].

The real numbers of irAEs are very difficult to estimate. On one hand, the incidence of rare irAEs is so low (encephalitis <1%, for example) that they cannot be easily identified or recognized as an irAE. On the other hand, for the most frequent irAEs, the challenge of identifying an accurate incidence is ascertaining the correct diagnosis. Many symptoms are nonspecific and are frequently confused with other clinical situations, such a infections, allergic reactions, or comorbidities [21]. In both situations, a late or wrong diagnosis can result in harm to the patient.

There are several factors that impact the frequency or site of irAEs. Depending on the antibody used, the incidence of side effects also varies. Colitis, hypophysitis, and rash are more common with CTLA-4 inhibitors, whereas pneumonitis, hypothyroidism, arthralgia, and vitiligo are more common with anti-PD-1 [22]. Distinct functions of ICI on immunoregulatory networks may explain these differences, but the precise mechanisms are unclear [23]. Apparently, there is no linear correlation between dose and toxicity with anti-PD-1/L1, in contrast to anti-CTLA-4 therapy, were the higher the dose the higher the rate of AEs [24, 25].

Considering the primary tumor type, collectively, the irAE data do not suggest any type of tumor-specific side effect [26]. Yet, when it comes to frequency, a systematic review, which analyzed 48 trials (6938 patients), has shown that different cancers that received the same ICI may present statistically significant differences in terms of frequency of development of certain irAEs. For example, patients with melanoma have a higher frequency of dermatological (mainly vitiligo) and gastrointestinal irAEs and a lower frequency of pneumonitis compared to non-small cell lung cancer and kidney cancer. The nature of this data (cross-study comparisons) does not allow us to confirm if these findings represent a true tumor pattern due to antigenic cross-reactivity or some type of bias [22].

Several investigations of risk factors for irAE development are ongoing and have covered factors such as genetic background, age, gender, body mass index, kidney function, and the influence of the microbiome [27–31]. Importantly, understanding why some patients do not develop irAEs after years of ICI therapy, and others may have life-threatening reactions after a single infusion is essential for further development in this field.

In addition to the concern about patient safety regarding irAEs, another crucial point is the possible impact on the effectiveness of the treatment. Earlier studies in melanoma suggested no association between irAEs and anti-CTLA-4 benefit [32]. In contrast, several retrospective studies have linked the development of irAEs due to anti-PD-1/L1 with improved response and survival. This relationship could be reflecting a more immunocompetent response or cross-reactivity between tumor and host tissue [33]. Interestingly, when analyzing these studies, the most consistent data which associates irAEs to ICI anti-tumor response are from dermatologic (particularly rash and vitiligo) and endocrine irAEs, leading to the hypothesis that the site of the appearance of irAEs may have importance [34].

Supporting the cross-reactivity theory, vitiligo is not a common side effect in patients with other cancers who received ICI, but up to 10% of melanoma patients can develop irAEs, which are associated with extremely high response rates (up to 70-80%) across immunotherapies in melanoma [35, 36]. Noteworthy, these same irAEs are the ones that in clinical practice do not require immunosuppression (hormonal replacement for the majority of endocrine irAE) and can be treated with topical, low dose, and short-term corticosteroids in most cases of dermatologic irAE [4, 37].

In contrast among non-small cell lung cancer patients who develop G3 or 4 pneumonitis and, as a consequence, receive high-dose corticosteroids for at least 4–6 weeks, tend to have a worse prognosis [38]. Here, one possibility is that therapy for pneumonitis may have caused the loss of the benefit achieved. This is a matter of debate that is complicated by heterogeneous scenarios, like the severity of irAE, the timing of onset (earlier or late), disease status (patients with complete response or with a high burden of active disease), dose, type (corticosteroids vs monoclonal anti-bodies, like infliximab), and duration of immunosuppressive treatment.

Retrospective studies have shown that outcomes for patients with melanoma who stopped treatment due to irAEs and used immune suppressant agents were not different from irAE-free patients who remained on treatment [16, 32, 39]. In contrast, one study of melanoma patients who developed hypophysitis while on treatment with ipilimumab (anti-CTLA-4) showed that those who received lower dose corticosteroids had substantially better survival compared to those treated with high-dose corticosteroids [40]. In patients with lung cancer treated with anti-PD-1 and anti-PD-L1 antibodies, those receiving corticosteroids at baseline had worse results than the ones not on corticosteroids [41].

Undoubtedly, there is a need for studies exploring potential relationships between these various aspects of immunosuppression, immune reactivation and ICI efficacy. There is also a need for predictive biomarkers that can identify those patients who will benefit from treatment, those who will present irAEs (and its severity), and those who will have both response and toxicity. Recently, strategies adding specific immunosuppressant monoclonal antibodies (e.g. anti-IL6, tocilizumab) to nivolumab (anti-PD-1) and ipilimumab (anti-CTLA-4) aiming at retaining efficacy and reducing toxicity have been initiated [42]. Identifying the ideal patient for these approaches and others, will bring important advances to this field.

## T-cell mediators of irAEs

### Immunoregulatory mechanisms

One of the great challenges for immuno-oncology today is to discover clinically actionable targets to inhibit the development of irAEs following ICI therapy, thereby preventing therapy interruption due to toxicity. The main blockers used clinically today inhibit the interactions between PD-1/PD-L1-PD-L2 or CTLA-4/CD28, all of which are essential for the normal control of exacerbated immune responses.

One of the first papers published by Tasuku Honjo’s group [43] demonstrated the importance of PD-1 as a negative immune regulatory receptor. In this study, performed in PD-1 deficient B6 mice, the lack of PD-1 induced a lupus-like glomerulonephritis and arthritis, associated with a strong inflammatory cell infiltration into many organs (epidermis, heart, lung, liver, etc.), demonstrating a breakdown of self-tolerance. In the context of irAEs, PD-1 blockade could induce reactivation and extra-tumoral inflammation mediated by autoreactive CD4+ and CD8+ T cells. Like the PD-1 deficiency, mice with a homozygous CTLA-4 mutation displayed severe disease pathology within 2–4 weeks. This was characterized by an increase of spleen and lymph node sizes caused by activation and proliferation of CD4+ and CD8+ T cells and B cells, as well as a high systemic infiltration of T cells in heart, lung and liver tissues, and myocardial infarctions (possibly causing death) [44].

Considering the difficulty of studying irAEs in preclinical models, research has focused on developing a model capable of mimicking this outcome. The FOXP3-DTR mouse model has emerged as an interesting model allowing for transient—one DT dose—or prolonged Treg depletion—multiple DT doses—to decrease the self-tolerance threshold. Prolonged Treg depletion demonstrated signs of illness like severe toxicity and required euthanasia, but a transient depletion demonstrated a better model to assess and study irAEs mechanisms following different treatments. In general, the irAEs were confirmed by lymphocytic mononuclear infiltration in many organs including colon and liver [45]. Mice that received one dose of DT and treated with anti-CD137 therapy demonstrated a pattern of inflammation (higher frequencies of CD8+ T cells and CD8+Ki67+ T cells and increased levels of IFN-γ and TNF-α) similar to a prolonged depletion, but not requiring euthanasia. The antitumor efficacy of different treatments (anti-PD-1/TIM-3/CD137) was also evaluated in this model, and anti-PD-1 or anti-TIM-3 demonstrated a better therapeutic window (high antitumor efficacy and low toxicities), while anti-CD137 was characterized by limiting toxicity [45].

Another model using B6/lpr mice to study immune infiltration in liver, colon, lung, and pancreas after PD-1 and CTLA-4 inhibition has also been used [46]. Like some clinical results, mice that develop irAEs (higher tissue immune infiltration) have a better response to treatment (lower tumor volume), pointing to shared antigens between tumor and normal tissues, or to the improvement of effector T cells with different specificities, including those with autoreactive potential. This model was also used to study the impact of treatment of irAE on the anti-tumor response, given steroid treatment (prednisolone) reduced autoimmune infiltration, but also reduce the anti-tumor activity [46]. Finally, a *Ctla4* gene humanized mouse model was used to study the development of irAEs after treatment with Ipilimumab or L3D10 (anti-CTLA-4 mAbs) and/or RMP1-14 (anti-PD-1 mAb) [47]. This model demonstrated a higher infiltration of CD45+ and CD3+ cells (CD4+ and CD8+ subsets) in the hearts, indicating T cell mediated pathology. To determinate the contribution of autoreactive T cells to irAE establishment, the authors evaluated anti-endogenous viral superantigen (VSA)-specific T cells and the impact of combined therapy on VSA-reactive effector or regulatory T cells in a mouse model capable of presenting endogenous superantigens (H-2d+ CTLA4h/h mice). Anti-CTLA-4 + anti-PD-1 treatment increased the frequency of autoreactive T cells and reduced Treg cell frequency among autoreactive T cells (reduced ratio Treg/Teff) [47]. Moreover, these studies suggested the potential compartmentalization of activating and differentiating autoreactive T cells versus antitumor T cells, as well as a differential transition into Treg cells.

Current studies have not fully identified specific mechanisms responsible for irAE development. However, the clinical management and potential mechanisms are based on those relevant to other pathologies such as autoimmunity and inflammatory disease [48]. In light of these diseases, the breakdown of self-tolerance induced by ICI could lead to differentiation and expansion of autoreactive T cells [49] and consequently promote an immune response targeting healthy tissues. The constitutive expression of CTLA-4 in pituitary endocrine cells explains the occurrence of hypophysitis in patients receiving anti-CTLA-4 [50] and highlights the importance of this potential mechanism due to constitutive expression of checkpoints in normal tissues. Lastly, as described previously, the occurrence of irAEs in tissues that share antigens with the tumor supports the cross-reactivity theory [35, 36], indicating that immune system activation leads not only to tumor destruction but also normal tissues through recognition of common antigens.

### T cells in irAEs development and physiopathology

**Figure 2:**
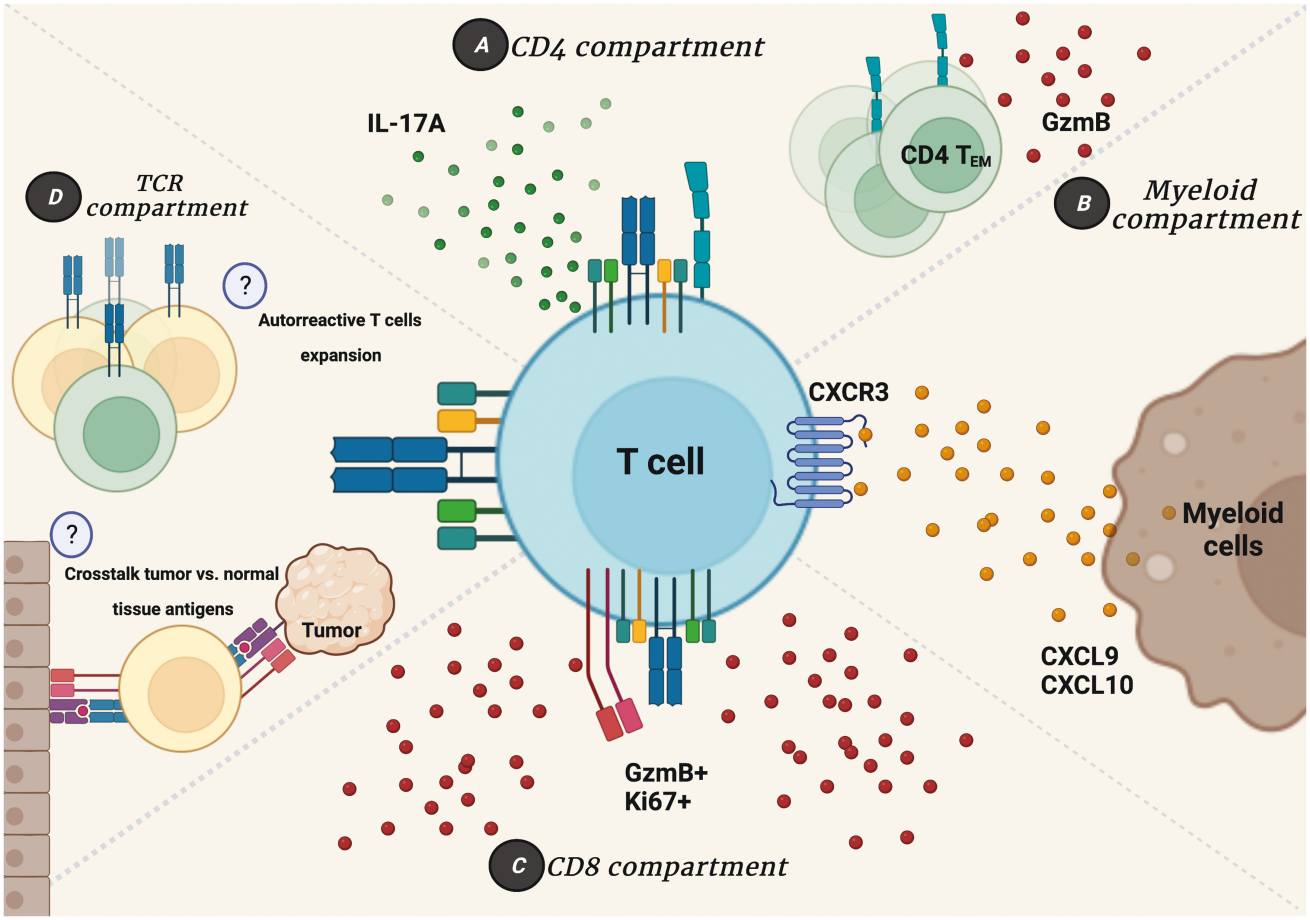
T-cell role in immune-related adverse events. The contribution of T cells to irAE development is the most studied in cancer immunotherapy toxicity induction, while some mechanisms are unclear or poorly understood. (A) CD4+ T-cell compartment highlights the contribution of pro-inflammatory subsets of CD4+ T cells producing IL-17A (characterizing an Th17 subtype), and also a subset of effector memory CD4+ T cells capable of producing Granzyme B and targeting normal tissues leading to inflammation and damage. (B) T cells also can contribute to irAEs when expressing receptors such as CXCR3 whose ligands CXCL9 and CXCL10 produced by myeloid cells allows an inflammatory response with migration of T cells to the normal tissue, Th1 polarization and consequently inflammatory establishment. (C) Many studies performed in mice and humans show the importance of CD8+ T cells on the irAEs site due to their proliferative capacity and granzyme B production that can target not only tumor cells but also normal tissue. (D) Biased T-cell receptor (TCR) repertoire distribution seems to be an important indicator of the antigen specific nature of irAE development. Some studies have shown an expansion of specific clonotypes; however, little is known about their specificity. Immunotherapy can induce the proliferation and differentiation of anti-tumor effector T cells and in some cases of autoreactive T cells whose TCR recognizes normal tissues antigens. In addition, some studies point to the possibility of a crosstalk between the tumor antigens and the normal tissues antigens, that can be recognized by T cells, so the anti-tumor response is directed also to these antigens presented in normal tissues that become inflamed and lead to irAEs. Figure designed using Biorender.com.

Studies using murine models pointed to the central role of T lymphocytes in the development of irAEs, suggesting the need to assess whether these same mechanisms are present in cancer patients treated with checkpoint inhibitors. NSCLC (non-small cell lung cancer) or TET (thymic epithelial tumor) patients treated with anti-PD-1, not exclusively as a first-line treatment, presented an increased frequency of activated CD8+ T cells (HLA-DR+CD38+) with a proliferative potential (Ki67+) that contributed to the increase of effector Treg cells [51]. The production of TNF-α and a higher Th17/Th1 ratio were associated with the development of severe irAEs. Furthermore, Treg and CD8+ Ki67+ T-cell subpopulations have an interplay in which Treg cells can compensate for the increased frequency of CD8+ Ki67+ T cells (Treg compensated). This profile has been a good parameter to predict the development of mild irAEs (grades G1 to G2) or, in case that the Treg subpopulation does not follow the increase in CD8+ Ki67+ T cells (Treg uncompensated) this parameter can predict the development of severe irAEs (grades G3 to G4) [51]. While this work brings an important discussion about potential mechanisms involved in irAE development, TET is a tumor frequently associated with autoimmune diseases. Also, the validation of these parameters in other tumors treated with immune-checkpoints inhibitors will be interesting to determine if the findings are generalizable to other tumors or are tumor dependent (Fig. 2).

The mechanisms underlying irAE establishment likely shares some common features with inflammatory and autoimmune disease mechanisms. The interplay between effector/cytotoxic subpopulations and regulatory cells has been reported previously in chronic graft-versus-host disease, systemic lupus erythematousus and others. One consequence of allogenic hematopoietic stem cell transplantation (HSCT) is the development of a chronic graft-versus-host disease where the patients who developed GVHD had decreased levels of Treg/Tconv (CD4) and Treg:CD8 ratios indicating an unfavorable regulatory scenario [52]. Supporting the findings of a disbalance of cellular subpopulations in GVHD and irAE’s [51], studies in the murine model of lupus erythematous showed a partial deficiency of peripheral Treg cells coupled with IFN-γ production by conventional CD4+ T cells [53].

A case report of a melanoma patient treated with Ipilimumab (development of colitis), targeted therapies (Vemurafenib, Dabrafenib and Trametinib) and Pembrolizumab (development of fulminant neurologic toxicity and meningoencephalitis) proposed other markers associated with AEs. Among all the markers, the expression of Ki67, CD45RO and GzmB by T cells shows the importance of activated memory T cells for toxicity of these treatments (Fig. 2) [54]. GzmB was associated with increased interstitial inflammatory infiltrate induced by checkpoint blockade in other tumor types [55, 56]. Moreover, a higher degree of T-cell clonality was also involved with tissue immune infiltration. Analysis of TCR repertoire and HLA typing highlighted TCR usage with similarity to EBV-specific TCRs, and the EBV TCR like was expressed by CD4+ T cells expressing Ki67, CD45RO, and GzmB [51]. The treatment with a combination of Ipilimumab and Nivolumab triggered fulminant myocarditis in melanoma patients [57] with an infiltration of CD4+ and CD8+ T cells, as well as macrophage infiltration, but not B-cell infiltration or antibody depositions, in the myocardium, skeletal muscle or tumor. Specific clones expanded in the myocardium and skeletal muscle also expanded in the tumor, highlighting the possibly that the same T-cell clones that recognize the tumor could recognize similar epitopes in a normal tissue, and thus, directing the irAE development after checkpoint inhibition.

The idea that clonal expansion of autoreactive T cells could be correlated with irAE development suggested in case reports was confirmed by other studies [49, 56, 58]. In addition to the finding that T-cell clonal expansion did not correlate with better outcome at baseline, the clonal expansion of CD8+ T cells posttreatment was increased in G2/G3 patients versus G0/G1 ones. Here, it is important to note that the posttreatment clonal expansion was not correlated with a clinical benefit, while it was correlated with irAEs development [58]. A recent study using a large cohort of metastatic melanoma case report results [54, 56, 57] demonstrated the importance of activated memory CD4+ T cells and also the importance of an early T-cell clonal expansion [59]. These findings suggest that in addition to a potential biomarker, the characterization of T cells involved in clonal expansion and their functional activity are crucial to separate potential mechanisms associated with response to treatment or development of AEs (Fig. 2).

## Role of B cells in irAEs

The participation of B lymphocytes in the anti-tumoral response, as well as in the development of AEs is less well studied. Although specific B lymphocyte subpopulations were not related to the development of irAEs, the production of autoreactive antibodies seems to be related [60–63]. When the target of these autoantibodies was evaluated, an enrichment of antibodies that recognize proteins related to apoptosis, TNF-α-signaling and IL-1 pathways were identified, which were also predictive of irAE development [61]. Other studies demonstrated that autoantibodies were associated mainly with thyroid dysfunction (hypothyroidism, thyrotoxicosis, and thyroiditis), one of the most frequent irAEs [62, 63].

## Role of myeloid cells in irAEs

Myeloid cells directly associated with the development of AEs have not yet been described [56, 64]; however, they can produce chemokines and cytokines capable of attracting inflammatory T cells, which are associated with toxicity mediated by blocking immune checkpoints. Myeloid cells from colitis patients were upregulated for IFN-γ inducible genes, such as CXCL9 and CXCL10, that are ligands of CXCR3, also upregulated in some CD4+ T cells clusters, indicating the potential recruitment of T cells to sites of irAEs [56]. Although this axis is could present an obstacle in irAEs management due to their important contribution to anti-tumor activity, it is important to understand the contribution of each ligand individually to driving ICI induced tumor killing versus tissue inflammation and irAE development.

## Future directions and challenges

While ICI therapy has dramatically increased survival and the quality of life for many patients with metastatic cancer, there are several key points that need to be resolved before expanding the clinical benefit even further. One is further research to identify biomarkers capable of predicting which patients will respond to a given ICI therapy, and the other is identification of biomarkers predictive of severe irAE development. With the discovery of these two classes of predictive biomarkers, it would be possible to greatly increase the overall benefit of ICI for patients with cancer, as well as lower costs due to better targeting of this costly therapy to those that would most benefit. This is a major factor in resource constrained countries where ICI therapy is generally not available for use by patients in the public health care system due to prohibitive costs. Some critical clinical questions that remain concerning irAEs and ICI therapy are:

- Can we personalize the individual patient risk? Who will develop irAEs, in what organ or system, and at which severity?
- Can we decrease this risk?
- Can we pharmacologically block irAEs without compromising therapeutic efficacy?

In conclusion, further studies focused on the mechanisms of irAEs establishment and validation of biomarkers capable of predicting toxicity development need to be performed in order to answer these questions and lead to an early risk classification of patients and also a specific management focusing on both response to therapy and irAEs development.

## Data Availability

Not applicable.

## Abbreviations

CTLA-4: cytotoxic T-lymphocyte-associated protein 4
DT: diphtheria toxin
GVHD: graft versus host disease
GzmB: granzyme B
HLA: human leukocyte antigen
ICI: immune checkpoint inhibitors
irAE: immune-related adverse event
PD1: programmed cell death protein 1
TCR: T-cell receptor
Th: T helper cells
